# The Neuroimaging Data Model Linear Regression Tool (nidm_linreg): PyNIDM Project

**DOI:** 10.12688/f1000research.108008.1

**Published:** 2022-02-24

**Authors:** Ashmita Kumar, Albert Crowley, Nazek Queder, JB Poline, Satrajit S. Ghosh, David Kennedy, Jeffrey S. Grethe, Karl G. Helmer, David B. Keator

**Affiliations:** 1 Troy High School, Fullerton, California, USA; 2 TCG, Inc., Washington, DC, USA; 3 Psychiatry and Human Behavior, University of California, Irvine, Irvine, California, USA; 4 Department of Neurology and Neurosurgery, McConnell Brain Imaging Centre, McGill University, Faculty of Medicine and Health Sciences, Montreal, Canada; 5 McGovern Institute for Brain Research, Massachusetts Institute of Technology, Cambridge, Massachusetts, USA; 6 Department of Otolaryngology, Harvard Medical School, Boston, Massachusetts, USA; 7 Department of Psychiatry, Eunice Kennedy Shriver Center, University of Massachusetts Chan Medical School, Worcester, Massachusetts, USA; 8 Department of Neuroscience, Harvard Medical School, San Diego, California, USA; 9 Department of Radiology, Massachusetts General Hospital, Harvard Medical School, Boston, Massachusetts, USA

**Keywords:** Linear Regression, Neuroimaging, PyNIDM, Neuroimaging Data Model, Machine Learning

## Abstract

The Neuroimaging Data Model (NIDM) is a series of specifications for describing all aspects of the neuroimaging data lifecycle from raw data to analyses and provenance. NIDM uses community-driven terminologies along with unambiguous data dictionaries within a Resource Description Framework (RDF) document to describe data and metadata for integration and query. Data from different studies, using locally defined variable names, can be retrieved by linking them to higher-order concepts from established ontologies and terminologies. Through these capabilities, NIDM documents are expected to improve reproducibility and facilitate data discovery and reuse. PyNIDM is a Python toolbox supporting the creation, manipulation, and querying of NIDM documents. Using the query tools available in PyNIDM, users are able interrogate datasets to find studies that have collected variables measuring similar phenotypic properties. This, in turn, facilitates the transformation and combination of data across multiple studies.

The focus of this manuscript is the linear regression tool which is a part of the PyNIDM toolbox and works directly on NIDM documents. It provides a high-level statistical analysis that aids researchers in gaining more insight into the data that they are considering combining across studies. This saves researchers valuable time and effort while showing potential relationships between variables. The linear regression tool operates through a command-line interface integrated with the other tools (pynidm linear-regression) and provides the user with the opportunity to specify variables of interest using the rich query techniques available for NIDM documents and then conduct a linear regression with optional contrast and regularization.

## Introduction

### Background

The Neuroimaging Data Model (NIDM) ( Keator *et al.* 2013; NIDM Working Group; Maumet *et al.* 2016) ( Neuroimaging Data Model, RRID:SCR_013667) was started by an international team of volunteers to create specifications for describing all aspects of the neuroimaging data lifecycle. NIDM is built upon the PROV Standard ( Moreau *et al.* 2008; “PROV-Overview”). It consists of three specifications: Experiment, Results, and Workflow. Using sematic web techniques ( “Semantic Web - W3C”), these specifications were envisioned to capture information on all aspects of the neuroimaging data lifecycle, producing graphs linking each result’s artifact with the workflow that produced it and the data used in the computation. These graphs can be serialized into a variety of text-based formats (NIDM documents), and with the capabilities of the semantic web, can be used to link datasets together through annotations with terms from formal terminologies, complete data dictionaries of study variables, and linkage of study variables to broader concepts. These annotations provide a critical capability to aid in reproducibility and replication of studies, as well as data discovery in shared resources. The NIDM-Experiment model consists of a simple project-session-acquisition hierarchy which can be used to describe both the content and metadata about experimental studies and derived (e.g., regional brain volume, mass-univariate functional brain analysis) neuroimaging data. It has been used to describe many large publicly-available human neuroimaging datasets (e.g. ABIDE ( Di Martino *et al.* 2014), ADHD200 ( Milham *et al.* 2011), CoRR ( Zuo *et al.* 2014) ( Consortium for Reliability and Reproducibility, RRID:SCR_003774), OpenNeuro ( “OpenNeuro”) ( OpenNeuro, RRID:SCR_005031) datasets) along with providing unambiguous descriptions of the clinical, neuropsychological, and imaging data collected as part of those studies.

PyNIDM ( *PyNIDM*) ( PyNIDM, RRID:SCR_021022) v3.9.5 is a Python toolbox under active development that supports the creation, manipulation, and query of NIDM documents. It is open-source and hosted on GitHub, distributed under the Apache License, Version 2.0 (“ Apache License, Version 2.0”). PyNIDM consists of tools to work with NIDM documents such as conversion from BIDS ( Gorgolewski *et al.* 2016), graph visualization, serialization format conversion, merging and query. Querying of NIDM documents is supported using a command-line RESTful ( Ravan *et al.* 2020) interface (i.e. pynidm query) which executes SPARQL ( “SPARQL Query Language for RDF”) queries. Using the query functionality and the NIDM document semantics, users can quickly identify datasets that measured similar properties and may be combined for further investigation.

Beyond the existing tools that have been written to support NIDM documents, some high-level statistical analysis tools are needed to provide investigators with an opportunity to gain more insight into data they may be interested in combining for a complete scientific investigation. Combining datasets collected across different studies is not a trivial task. It requires both a complete, unambiguous description of the data and how it was collected, along with a varying number of transformations to align, where possible, disparate data. The process of transforming data is often quite time-consuming and therefore understanding whether the identified datasets, at a high level, might have some interesting relationships prior to committing to a full scientific study is prudent. Here we report on a tool that provides such capabilities; namely, a simple linear modeling tool supporting NIDM documents and integrated into the existing PyNIDM suite of tools.

### Statement of need

While tools and libraries for statistics and machine learning algorithms are numerous, there are none that can be directly applied to NIDM documents. The linear regression algorithm presented here allows scientists studying the human brain to easily find relationships between variables across datasets while retaining the provenance present in NIDM documents. The algorithm has the ability to query for specific variables or across similar variables from different studies using concept annotations on the variables. It then provides the user with the ability to construct arbitrary linear models on those data, supporting interactions between variables, contrasts of learned parameter sets, and L1 and L2 regularization ( Nagpal 2017). There is no comparable tool for this use case.

## Methods

### Implementation and use cases

The linear regression tool, *nidm_linreg* , uses the PyNIDM query functionality to aggregate data in NIDM documents serialized using the standard Terse Resource Description Framework (RDF) Triple Language (TURTLE) ( “RDF 1.1 Turtle”), a common semantic-web serialization format that is both structured for ease of use with computers and relatively easy for humans to read. Researchers have the ability to construct custom models based on their scientific goals. The source code is available on Zenodo and full details can be found in the Software Availability statement ( Keator *et al.* 2021).

Thus, nidm_linreg is a machine learning algorithm that can work on complex datasets described using the NIDM linked-data format, while being reasonably easy to use. Researchers have the ability to conduct a preliminary analysis to understand if it is worth the effort to pursue combining datasets and doing the transformations necessary to integrate those datasets. One can quickly determine if there are high-level relationships in the datasets and look at the different weights to decide what variables may warrant further study.

**Figure 1.**
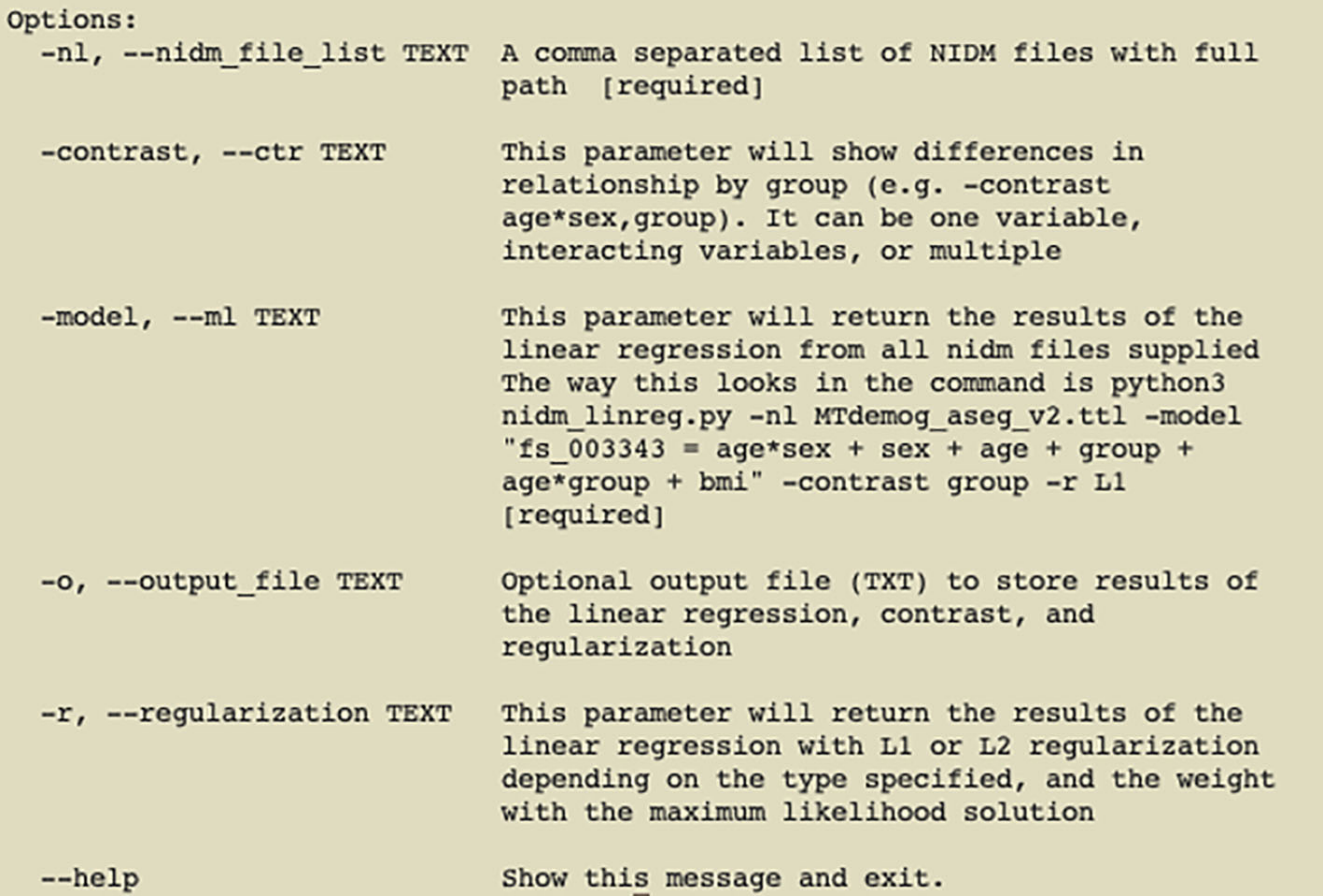
pynidm linear-regression parameters; demonstrating options for a researcher using the tool.

The tool provides a simple command-line user interface ( Figure 1) based on the “Click” Python library ( “Welcome to Click — Click Documentation (8.0.X)”) which integrates the linear regression module with existing PyNIDM tools (e.g. pynidm linear-regression, pynidm query, pynidm convert, etc.).

**Figure 2.**
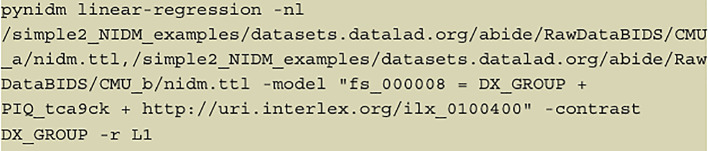
The full command; an example of a command the user can specify to begin the linear regression on selected variables, with regularization at the end.

To use the tool, the user runs the command pynidm linear-regression with a variety of required and optional parameters. The first parameter, “-nl”, is a comma- separated list of NIDM serialized TURTLE files, each representing a single dataset or a collection site within a multi-site research project or multiple datasets ( Figure 2). A useful set of NIDM documents describing publicly-available neuroimaging data from the ABIDE, ADHD200, and CoRR studies along with datasets in the OpenNeuro database can be found on GitHub ( D. Keator) The next parameter, “-model” provides the user with the ability to construct a linear model using notation found in popular statistics packages (e.g., R statistical software ( Ripley 2001) ( R Project for Statistical Computing, RRID:SCR_001905)). The syntax follows the scheme “dependent variable (DV) = independent variable 1 (IV1) + independent variable 2 (IV2) + … + IVX”. To encode interactions between IV1 and IV2 in the above example, one can use the common “*” syntax: “DV = IV1 + IV2 + IV1*IV2”.

To determine what variables or data elements are available from a set of NIDM documents, the first step is to use “pynidm query” to do a data element search of the NIDM documents. From this search, the user can see what data elements are available in the selected NIDM documents and understand some details of those data elements (e.g., ranges, categories, data type, etc.). After performing the data elements query of the NIDM documents and selecting independent and dependent variables of interest, one proceeds with constructing the linear model with the pynidm linear-regression tool.

In the example shown in Figure 2, we have first run a pynidm query operation on the NIDM documents and identified four variables of interest: supratentorial brain volume (fs_000008), diagnostic group (DX_GROUP), performance IQ (PIQ_tca9ck), and age. The model specified establishes the relationship between the DV, brain volume, and the IVs, diagnostic group, performance IQ, and age. In this example, fs_000008 is the fixed unique identifier (UUID) of the supratentorial brain volume computed with the FreeSurfer software ( Fischl 2012) ( FreeSurfer, RRID:SCR_001847) using the original Magnetic Resonance Imaging (MRI) structural scans of the brain. This particular UUID is fixed because it identifies a specific brain region and measurement computed with the FreeSurfer software and will not change across datasets that derive brain volume measurements with FreeSurfer. DX_GROUP is the name of the study-specific variable describing the diagnostic group assigned to participants. PIQ_tca9ck is the performance IQ measure collected on study participants and is the UUID created for this data element when the NIDM documents were created for this dataset. Note, this particular UUID is not guaranteed to be the same across NIDM documents from different studies. Finally, “ http://uri.interlex.org/ilx_0100400” is the age of the participants using a URL form to reference a concept describing the high-level measure of age which has been used to annotate the variables measuring age across studies. Here we use a concept URL that has been mapped to each dataset’s separate variables that store the age of participants. By using the concept URL, we avoid the complexity of different variable names being used to store consistent information (e.g., age) across datasets.

This example shows that one can select data elements from the NIDM documents for linear regression using three specific forms: (1) using the UUID of the objects in the NIDM graph documents; (2) using the distinct variable name from the original dataset, also stored as metadata in the NIDM graph documents; (3) using a high-level concept that has been associated with specific variables described by the concept across datasets, used to make querying across datasets with different variable names but measuring the same phenomenon easier. We support these three distinct forms of selecting data elements to enable distinct usage patterns. Some investigators will use NIDM documents of their internal studies and want to be able to reference data elements using their study-specific variable names. Other investigators may want to use variables from different studies and thus the variable names are unlikely to be the same; thus, we support the use of selecting variables based on high-level concepts. In practice, users will not often mix forms of referring to data elements within the same model, but we show it here to make evident the flexibility of the tool.

**Figure 3.**
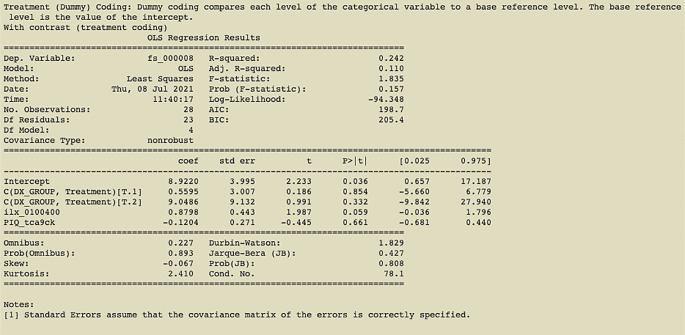
Output of command in Figure 2 with treatment coding (contrast using diagnostic group); an example of the printout given after linear regression analysis is complete.

The optional “-contrast” parameter allows one to select one or more IVs to contrast the parameter estimates for those IVs. The contrast variable in this example is “DX_GROUP” which describes the diagnostic group of each participant. Our tool supports multiple methods of coding treatment variables (e.g., treatment coding ( Figure 3), simple coding, sum coding, backward difference coding, and Helmert coding) as made available by the Patsy Python library ( Brooke 1923). The user can select multiple independent variables to contrast and/or contrasts on interactions. The results of the treatment coding contrast applied in Figure 2 can be seen in Figure 3.

The optional “-r” parameter allows the user to select L1 (Lasso) or L2 (Ridge) regularization implemented in scikit-learn ( Varoquaux *et al.* 2015) ( scikit-learn, RRID:SCR_002577). In either case, regularizing prevents the data from being overfit, potentially improving model generalizability and demonstrating which variables have the strongest relationships with the dependent variable. The regularization weight is iteratively determined across a wide range of regularization weightings using 10-fold cross-validation, selecting the regularization weight yielding the maximum likelihood.

There are error checks within the code to make sure the researcher has feedback on why a model cannot run, whether it is because there are not enough data points or because one or more variables could not be found in one or more of the NIDM documents. This makes the experience as simple as possible for the user, which is important, as our intended audience for these tools are investigators who may have no prior experience with the semantic web and/or NIDM documents.

**Figure 4.**
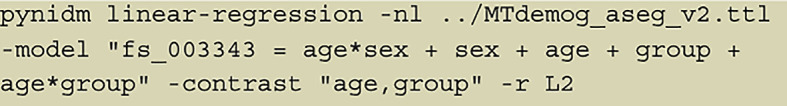
Model command with interacting variables and multivariable contrast; an example of another command the user can specify to conduct a linear regression analysis with interactions encoded and a multivariable contrast.

In the example shown in Figure 4, we have first run a pynidm query operation on the NIDM documents and identified 4 variables of interest: fs_003343, age, sex, and group. Here, fs_003343 is the fixed unique identifier (UUID) of the left hippocampus volume while age, sex, and group are names of the study-specific variables concerning the age of the participant at the time of the study, the gender, and the group the participant was in. The model specified establishes the relationship between the DV, left hippocampus volume, and the IVs, group, age, and sex. However, in this case, we also encode interactions between age and sex and age and group, as denoted by the asterisks. Also, in this model, we have used multiple IVs to contrast the parameter estimates for those IVs. The contrast variables are age and group. Finally, L2 regularization is selected for regularization.

**Figure 5.**
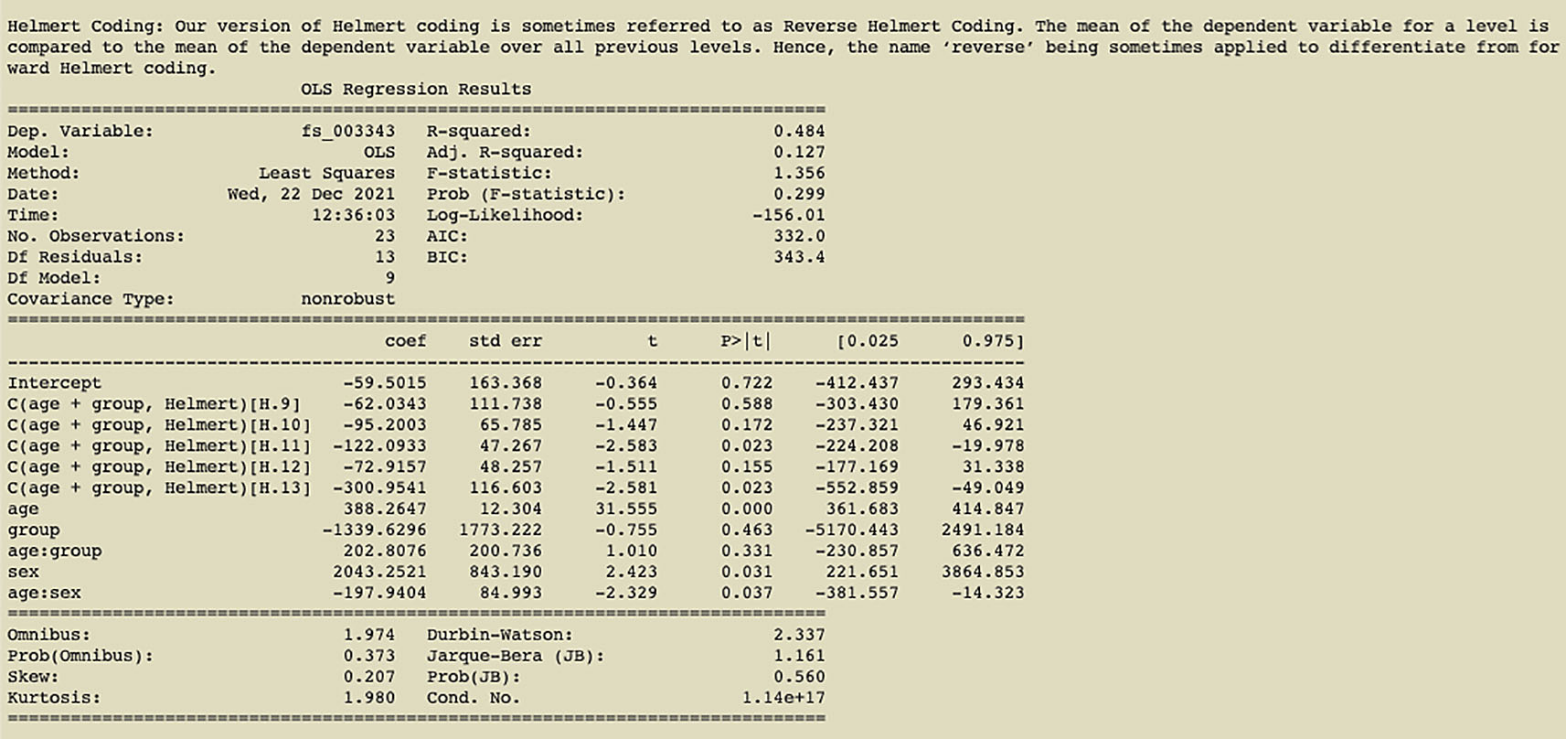
Output of command in Figure 4 with Helmert coding (contrast using age and group); an example of the printout given after linear regression analysis is complete.

The results of the Helmert coding contrast can be seen in Figure 5.

## Operation

The data must be in a NIDM document(s) to be used with this tool. Data can be transformed into a NIDM document directly from BIDS or tabular data files using the PyNIDM tools “bidsmri2nidm” and “csv2nidm”. Once data is transformed into NIDM, the user only needs to have a functional installation of PyNIDM and access to a terminal window or similar command-line processing tool with a functional version of Python 3. Once the user specifies the parameters, data is aggregated from the NIDM files, re-structured for the linear regression algorithm, and parameter estimates learned using ordinary least squares and returning either a printout or output file of the various coefficients and summary statistics.

## Conclusions

In this work, we have designed a linear regression tool that works on linked-data NIDM documents in support of understanding relationships between variables collected across studies. This tool helps scientists evaluate relationships between data prior to fully integrating datasets for hypothesis testing which may require considerable time and resources. In our initial evaluations, this tool has shown utility for these use cases. In future work, we are creating additional machine learning tools allowing users to cluster data in a similar fashion to the linear regression tool presented here. Further, the NIDM community is working on additional functionality for the PyNIDM toolkit that transforms the value representations of the variables selected for modeling to be consistent across all NIDM documents used in the model. These transformations are made using the detailed data dictionaries included in the NIDM documents. This functionality will be included in the PyNIDM query application programmers’ interface (API) and will be immediately available to the linear regression tool presented here.

## Software availability

Source code available from: https://github.com/incf-nidash/PyNIDM/blob/master/nidm/experiment/tools/nidm_linreg.py

Archived source code at time of publication: v3.9.5, https://doi.org/10.5281/zenodo.4635287 ( Keator *et al.*, 2021)

License: Apache License, Version 2.0
