# Supplementary material for: The Neuroimaging Data Model Linear Regression Tool (nidm_linreg): PyNIDM Project
Source: F1000Res. 2022 Jul 29;11:228. Originally published 2022 Feb 24. [Version 2] doi: 10.12688/f1000research.108008.2 (PMC11344200; doi:10.12688/f1000research.108008.2)
Supplement: Peer review history

# Reviewer report: Reviewer response for version 2

**DOI:** 10.5256/f1000research.136808.r277204

**Published:** 2024-08-23

**Authors:** Gabriele Lohmann

**Affiliations:** 1 Max Planck Institute for Biological Cybernetics, Tübingen, Germany

The paper under review presents the PyNIDM project, which has successfully developed a Python-based toolbox for regression analysis. This toolbox is built upon the Neuroimaging Data Model (NIDM), a well-established framework within the neuroimaging community. The primary focus of the paper is on a linear regression tool that leverages high-level representations, enabling the integration of data from multiple studies, even when these studies use diverse, locally defined variable names. The tool facilitates mapping these variables to higher-order concepts derived from established ontologies.

 The concept underpinning the NIDM and the regression tool is both innovative and commendable, offering significant potential to advance the field by promoting interoperability and the reuse of data across studies.

 However, while the high-level functionality provided by this tool is impressive, there are important considerations that must be addressed. One potential concern is that the use of such high-level tools could inadvertently lead researchers to overlook the complexities and challenges associated with noisy data. Specifically, the tool does not incorporate quality control measures or account for the nuances of harmonizing data obtained from different scanners, which may have employed varying acquisition parameters. In my experience, these factors can be critical, and failing to account for them could result in erroneous or biased outcomes.

 Perhaps the authors may want to add a word of caution reminding researchers to actually look at the data before blindly applying a high-level tool.

Are the conclusions about the tool and its performance adequately supported by the findings presented in the article?

Yes

Is the rationale for developing the new software tool clearly explained?

Yes

Is the description of the software tool technically sound?

Yes

Are sufficient details of the code, methods and analysis (if applicable) provided to allow replication of the software development and its use by others?

Yes

Is sufficient information provided to allow interpretation of the expected output datasets and any results generated using the tool?

Yes

Reviewer Expertise:

Development of mathematical methods for the analysis of magnetic resonance images.

I confirm that I have read this submission and believe that I have an appropriate level of expertise to confirm that it is of an acceptable scientific standard.

# Reviewer report: Reviewer response for version 2

**DOI:** 10.5256/f1000research.136808.r245708

**Published:** 2024-02-23

**Authors:** Tengfei Li

**Affiliations:** 1 The University of North Carolina at Chapel Hill, Chapel Hill, North Carolina, USA

The rationale for developing the new software tool has clearly been explained. The challenge of statistical modeling on NIDM has been generally explained. The workflow of the software has been generally described. However, there still exist some problems. 

 1) No comparison to any other similar tools. Are there any similar statistical/AI packages from software python, R, Matlab or SAS. A comparison can be made in their convenience, computational speed, and consistency of the results.

 2) No validation of the results through some simulated dataset to ensure accuracy.

Are the conclusions about the tool and its performance adequately supported by the findings presented in the article?

Partly

Is the rationale for developing the new software tool clearly explained?

Yes

Is the description of the software tool technically sound?

Yes

Are sufficient details of the code, methods and analysis (if applicable) provided to allow replication of the software development and its use by others?

No

Is sufficient information provided to allow interpretation of the expected output datasets and any results generated using the tool?

Yes

Reviewer Expertise:

Biostatistics

I confirm that I have read this submission and believe that I have an appropriate level of expertise to confirm that it is of an acceptable scientific standard, however I have significant reservations, as outlined above.

# Reviewer report: Reviewer response for version 2

**DOI:** 10.5256/f1000research.136808.r145980

**Published:** 2022-09-20

**Authors:** Adam G. Thomas, Eric Earl

**Affiliations:** 1 Data Sharing and Science Team, National Institute of Mental Health, Bethesda, MD, USA

The authors have provided detailed responses to points 1, 2, 4, and 5 in our initial review. However, point #3 and the minor point #6 were not addressed. 

 Regarding point #3, the commands shown in the screenshots in Figure 2 and Figure 4 still fail in our hands. We are now providing the error messages we received when attempting to run these commands (see below). Before resubmitting this manuscript, we suggest the authors make it easier for readers to run the two use cases described in the paper (perhaps by providing script or a container) and ask a few colleagues without PyNIDM experience to perform a fresh install of the tool and run the commands for each use case. 

 Regarding point #6, the “Patsy” article from 1923 is still listed in the references as “Brooke MC” and in the paper as “Brooke 1923”:

 Brooke MC: Patsy. The Psychological Clinic 1923; 15(1-2): 41–43. PubMed Abstract

 However, this is still not a reference for the Patsy Python Library.

**Additional minor points **

1. In the software availability section, the authors list the source code as available from a GitHub URL that will not necessarily reflect the code at the time of publication:
https://github.com/incf-nidash/PyNIDM/blob/master/nidm/experiment/tools/nidm_linreg.py
  We suggest tagging the version used in the publication and sharing a URL using that tag something like:
https://github.com/incf-nidash/PyNIDM/blob/v3.9.7/nidm/experiment/tools/nidm_linreg.py
2.  In the software availability section, the “archived source code” link ( https://zenodo.org/record/4635287#.Yxdu1HbMKHs) points to:
  incf-nidash/PyNIDM: PyNIDM tools v3.7.6 (dev) rather than the version that is referenced in the paper: version 3.9.7. The authors should update the text and the version uploaded to Zenodo.
3. The authors might consider closing PyNIDM GitHub issue 309 ( https://github.com/incf-nidash/PyNIDM/issues/309) with a comment referencing and explaining the commit used for resolving the issue.

**Errors & troubleshoot for commands in Figures 2 and 4**

 In our hands, the Figure 2 command fails with the following output:

***********************************************************************************************************Your command was: pynidm linear-regression -nl simple2_NIDM_examples/datasets.datalad.org/abide/RawDataBIDS/CMU_a/nidm.ttl,simple2_NIDM_examples/datasets.datalad.org/abide/RawDataBIDS/CMU_b/nidm.ttl -model "fs_000008 = DX_GROUP + PIQ_tca9ck + http://uri.interlex.org/ilx_0100400" -contrast "DX_GROUP" -r L1

***********************************************************************************************************

*Your model was fs_000008 = DX_GROUP + PIQ_tca9ck + http://uri.interlex.org/ilx_0100400*

*The following variables were not found in simple2_NIDM_examples/datasets.datalad.org/abide/RawDataBIDS/CMU_a/nidm.ttl. The model cannot run because this will skew the data. Try checking your spelling or use nidm_query.py to see other possible variables.*

*1. PIQ_tca9ck*

***********************************************************************************************************

*Your model was fs_000008 = DX_GROUP + PIQ_tca9ck + http://uri.interlex.org/ilx_0100400*

*The following variables were not found in simple2_NIDM_examples/datasets.datalad.org/abide/RawDataBIDS/CMU_b/nidm.ttl. The model cannot run because this will skew the data. Try checking your spelling or use nidm_query.py to see other possible variables.*

*1. PIQ_tca9ck*

 We searched for the simple2_NIDM_examples using Google and obtained the NIDM examples files from the github repository here:  https://github.com/dbkeator/simple2_NIDM_examples

 Upon inspection of the TURTLE files using pynidm, their PIQ files do not appear to match and neither refer back to “PIQ_tca9ck”:

*> pynidm query -nl simple2_NIDM_examples/datasets.datalad.org/abide/RawDataBIDS/CMU_a/nidm.ttl -de | grep PIQ                                                                                                                            30                              http://iri.nidash.org/PIQ_20o78sc  http://purl.org/nidash/nidm#PersonalDataElement* *31                    http://iri.nidash.org/PIQ_TEST_TYPE_1gkp148  http://purl.org/nidash/nidm#PersonalDataElement*

*> pynidm query -nl simple2_NIDM_examples/datasets.datalad.org/abide/RawDataBIDS/CMU_b/nidm.ttl -de | grep PIQ*

*30                              http://iri.nidash.org/PIQ_1g51r81  http://purl.org/nidash/nidm#PersonalDataElement*

*31                    http://iri.nidash.org/PIQ_TEST_TYPE_1mjbgt1  http://purl.org/nidash/nidm#PersonalDataElement*

 The command in Figure 4 fails with a "No such file" error as the "MTdemog_aseg_v2.ttl" file is not present in the simple2_NIDM_examples repository. A google search of this file name returned zero hits.

***************************

Your command was: pynidm linear-regression -nl ./simple2_NIDM_examples/MTdemog_aseg_vs.ttl -model "fs_003343 = age*sex + sex + age + group +age*group" -contrast "age,group" -r L2

*Traceback (most recent call last):*

*[snip]*

*FileNotFoundError: [Errno 2] No such file or directory: './simple2_NIDM_examples/MTdemog_aseg_vs.ttl'*

Are the conclusions about the tool and its performance adequately supported by the findings presented in the article?

Partly

Is the rationale for developing the new software tool clearly explained?

Yes

Is the description of the software tool technically sound?

Yes

Are sufficient details of the code, methods and analysis (if applicable) provided to allow replication of the software development and its use by others?

No

Is sufficient information provided to allow interpretation of the expected output datasets and any results generated using the tool?

Yes

Reviewer Expertise:

Neuroimaging, Neuroscience, Computer Science, Data Science

We confirm that we have read this submission and believe that we have an appropriate level of expertise to state that we do not consider it to be of an acceptable scientific standard, for reasons outlined above.

# Response

**Published:** 2022-10-06

**Authors:** Ashmita Kumar

**Affiliations:** Troy High School/University of California, Irvine, USA

Thank you for your feedback on our tool. We appreciate your thoroughness and will ensure to make the required changes. To your first point, we wanted to ask: do the commands work when you use https://github.com/AKUMAR0019/simple2_NIDM_examples instead of the https://github.com/dbkeator/simple2_NIDM_examples link? We updated it with the examples used in our figures, which will hopefully solve the issue. It worked for us when we did a clean install, but we want to double check before we update the link in the next version. To your second point, we will be sure to find the proper citation for the Patsy Python Library, and we apologize for not fixing it in the last version. We will be sure to address it. To your third point, we will make sure to put the appropriate tag on all our references to the source code, and are currently working on the Zenodo issue. To your fifth point, we will close the issue on GitHub.

 Thank you for taking the time to make this review, and we are sorry for the length of time it has taken to respond. We really appreciate it.

# Response

**Published:** 2023-01-13

**Authors:** Ashmita Kumar

**Affiliations:** Troy High School/University of California, Irvine, USA

Hi,

 Thank you for your time and your patience. We would just like to let you know that we are still resolving the link issue with Zenodo before we publish another version. However, we hope you had a chance to view our prior comment, and we are ready to respond to any questions or other feedback you may have.

 We appreciate your thorough review.

# Reviewer report: Reviewer response for version 1

**DOI:** 10.5256/f1000research.119281.r125519

**Published:** 2022-04-11

**Authors:** Karsten Specht

**Affiliations:** 1 Department of Biological and Medical Psychology, University of Bergen, Bergen, Norway

In this software tool article, the authors describe a tool that allows high-level statistical analysis for combining studies that follow the Neuroimaging Data Model (NIDM) framework. The tool is/will be part of the PyNDIM toolbox.

 In general, this appears as a very useful tool that a larger community might want to use. However, the article is phrased and organized in a way that this might not happen. The audience of the current manuscripts is almost exclusively the NDIM community that is involved in developing the tools, but it is not directed to potential users. The main problem is, in my view, that the level of explanation and description of the tool is very minimalistic. Of course, all important aspects and relevant tools are mentioned, but only as keywords with the appropriate references. My main concern is that readers not entirely familiar with all aspects and methods incorporated into this tool will not get its importance and relevance.

 I would like to see just a few more sentences describing the key elements in a revised version. For example, readers unfamiliar with the PROV standard or the semantic web technique are already lost after the third sentence. And the introduction continues like that. For example, the “L1 and L2 regularization” is just thrown in on the first page, assuming that every reader immediately gets its relevance, but a more relevant discussion follows much later.

 Similarly, I would have expected a more detailed description of the implementations in the section that is called “implementation and use cases”, but this section is mainly on “use cases” and very little on implementation.

 So, in summary, I think a revised version of this article would benefit from just a few more describing lines so that this very relevant tool generates interest in a broader audience.

Are the conclusions about the tool and its performance adequately supported by the findings presented in the article?

Yes

Is the rationale for developing the new software tool clearly explained?

Yes

Is the description of the software tool technically sound?

Yes

Are sufficient details of the code, methods and analysis (if applicable) provided to allow replication of the software development and its use by others?

Partly

Is sufficient information provided to allow interpretation of the expected output datasets and any results generated using the tool?

Yes

Reviewer Expertise:

Neuroimaging, methods development, reliability measures

I confirm that I have read this submission and believe that I have an appropriate level of expertise to confirm that it is of an acceptable scientific standard, however I have significant reservations, as outlined above.

# Response

**Published:** 2022-04-30

**Authors:** Ashmita Kumar

**Affiliations:** Troy High School/University of California, Irvine, USA

Thank you for the time you have taken to review this paper. We will add more specifics to make it more accessible to potential users, defining our implementation, data structures, and technique in more detail. We apologize for the time it has taken to respond, but rest assured we are working to address it in our next version.

# Reviewer report: Reviewer response for version 1

**DOI:** 10.5256/f1000research.119281.r125517

**Published:** 2022-04-08

**Authors:** Adam G. Thomas, Eric Earl

**Affiliations:** 1 Data Sharing and Science Team, National Institute of Mental Health, Bethesda, MD, USA

The authors have written about the nidm_linreg tool which is a linear regression tool that is now a part of the PyNIDM software. nidm_linreg was added because PyNIDM did not have a tool like this in its suite yet. The tool’s design and the paper’s focus on usage are both very thorough. This software tool article is likely of interest to PyNIDM and other members of the neuroimaging community. We have the following comments and concerns about the software and the paper:

1. Echoing the concern expressed in Dec 2021 in the PyNIDM GitHub issue 309 ( https://github.com/incf-nidash/PyNIDM/issues/309), it is bad practice to install Python packages outside of dependency management functionality. The nidm_linreg.py tool currently tries to quietly install 4 packages using pip if they are not present in the Python environment. This will likely cause problems and/or confusion for the user. A safe and reproducible way to provide the end-user with the dependencies they need is to provide a requirements.txt file with the repository which lets users use a specific version of the expected package dependencies. https://pip.pypa.io/en/stable/reference/requirements-file-format/
2. We ran the commands on the GitHub README.md (with some necessary modifications) to install and test PyNIDM (see below). pytest returned several errors and warnings (on two different platforms) that should be corrected:
  conda create -n pynidm_py3 python=3
  git clone https://github.com/incf-nidash/PyNIDM.git
  cd PyNIDM
  pip install -e .
  pytest
  [snip]
  = 4 failed, 24 passed, 14 skipped, 3 warnings, 36 errors in 35.41s ==
** **
3. Running the commands as-written in Figures 2 and 4 did not work. We cloned the simple2_NIDM_examples repository ( https://github.com/dbkeator/simple2_NIDM_examples) and tried to use the data within, but we were only able to run one of the example commands successfully.
  This felt insufficient to evaluate the reproducibility of the tool. The authors should provide commands, script, or a container such that users can reproduce the same outputs presented in the paper. 
4. The Methods section “Implementation and use cases” covers a blend of those two things where we think it would read better to see a focused section on Implementation and a focused section on Use cases. The Implementation section could discuss the design decisions and software architecture as well as planned support. Most of the current content could move over to a Use cases section covering a use case or two (as it does now).
5. The paper mentioned there are “error checks” built into the tool, but no examples are shown. Including a sentence about the kinds of errors being checked for would be another useful addition.
6. The reference for the Patsy Python Library (Brooke 1923).on page 5 of the PDF (Methods section, 8th paragraph) is incorrect.

Are the conclusions about the tool and its performance adequately supported by the findings presented in the article?

Partly

Is the rationale for developing the new software tool clearly explained?

Yes

Is the description of the software tool technically sound?

Yes

Are sufficient details of the code, methods and analysis (if applicable) provided to allow replication of the software development and its use by others?

No

Is sufficient information provided to allow interpretation of the expected output datasets and any results generated using the tool?

Yes

Reviewer Expertise:

Neuroimaging, Neuroscience, Computer Science, Data Science

We confirm that we have read this submission and believe that we have an appropriate level of expertise to state that we do not consider it to be of an acceptable scientific standard, for reasons outlined above.

# Response

**Published:** 2022-04-30

**Authors:** Ashmita Kumar

**Affiliations:** Troy High School/University of California, Irvine, USA

Thank you for your feedback on our tool. We appreciate your thoroughness and will ensure to make the required changes. To your first point, we will make sure that our package requirements are presented to the user before installation instead of using a quiet install. To your second point, while we are unsure why the error appeared, we are looking into it and attempting to reproduce it. We will be sure to address it in our next version. To your third point, we will make sure to provide a way for users to replicate the outputs in the paper by providing more specifics on how we obtained them. To your fourth point, we will separate the two sections and add to our Implementation section in our next version. To your fifth point, the error checks built in include data validation for type and quantity of that data, which we will explain in more detail. To your final point, we will try to find the correct citation.

 Thank you for taking the time to make this review, and we are sorry for the length of time it has taken to respond. We really appreciate it.
